# Flexible Margin Kinematics and Vortex Formation of *Aurelia aurita* and Robojelly

**DOI:** 10.1371/journal.pone.0098310

**Published:** 2014-06-06

**Authors:** Alex Villanueva, Pavlos Vlachos, Shashank Priya

**Affiliations:** 1 Center for Energy Harvesting Materials and Systems (CEHMS), Bio-Inspired Materials and Devices Laboratory (BMDL), Department of Mechanical Engineering, Virginia Polytechnic Institute and State University, Blacksburg, Virginia, United States of America; 2 Department of Mechanical Engineering, Purdue University, West Lafayette, Indiana, United States of America; University of California San Diego, United States of America

## Abstract

The development of a rowing jellyfish biomimetic robot termed as “Robojelly”, has led to the discovery of a passive flexible flap located between the flexion point and bell margin on the *Aurelia aurita*. A comparative analysis of biomimetic robots showed that the presence of a passive flexible flap results in a significant increase in the swimming performance. In this work we further investigate this concept by developing varying flap geometries and comparing their kinematics with *A. aurita*. It was shown that the animal flap kinematics can be replicated with high fidelity using a passive structure and a flap with curved and tapered geometry gave the most biomimetic performance. A method for identifying the flap location was established by utilizing the bell curvature and the variation of curvature as a function of time. Flaps of constant cross-section and varying lengths were incorporated on the Robojelly to conduct a systematic study of the starting vortex circulation. Circulation was quantified using velocity field measurements obtained from planar Time Resolved Digital Particle Image Velocimetry (TRDPIV). The starting vortex circulation was scaled using a varying orifice model and a pitching panel model. The varying orifice model which has been traditionally considered as the better representation of jellyfish propulsion did not appear to capture the scaling of the starting vortex. In contrast, the pitching panel representation appeared to better scale the governing flow physics and revealed a strong dependence on the flap kinematics and geometry. The results suggest that an alternative description should be considered for rowing jellyfish propulsion, using a pitching panel method instead of the traditional varying orifice model. Finally, the results show the importance of incorporating the entire bell geometry as a function of time in modeling rowing jellyfish propulsion.

## Introduction

Autonomous Underwater Vehicles (AUVs) such as the REMUS offer a wide range of applications but are limited by short operation lifetime ranging between a few hours to few days [1]. Vehicle self-sustainability consists of autonomous control, robustness and energy independence. Energy independence can be achieved by energy harvesting and increasing the vehicle efficiency. Several energy sources can be harvested in ocean waters such as wave, solar, thermal and chemical energy but more research is required to adequately exploit these resources in order to make any practical use for AUVs. Vehicle efficiency is therefore of paramount importance for reducing the amount of energy needed for sustaining the vehicle. More specifically, propulsion efficiency is critical when a vehicle must cover long distances or constantly propel itself to maintain a certain location. The propulsion system has three main sections where reducing losses becomes important: the actuators, the motion translating mechanism and the hydrodynamics. Biological systems have been able to significantly minimize the losses and achieve efficiencies higher than any engineered system. We therefore take inspiration from biology for answering the questions related to vehicle efficiency and for determining fundamental principles that can lead towards self-sustainable AUVs.

Jellyfish can be separated into two categories based on their mode of propulsion, namely: jetting and rowing [2]. Jetting is utilized by smaller species of prolate medusa generally not exceeding a few centimeters in diameter. This method of propulsion is known for higher velocities and accelerations [3]. Rowing is found in larger jellyfish reaching up to 2 m in diameter which are more oblate in geometry. This is a more efficient mode of propulsion [2]. *Aurelia aurita* fall in the category of rowers. The swimming mechanism for most jellyfish consists of circular muscles located in the subumbrella which collapses the flexible bell upon contraction. The collapse of the bell causes a volume change in the subumbrella which leads to the expulsion of water and thrust production. The relaxation phase is driven by the elastic energy stored in the bell structure during contraction [4]–[8]. For rowers, a stopping vortex is formed under the bell during relaxation. This is followed by a starting vortex formed during contraction. The starting and stopping vortices interact with each other to increase thrust [2].

Recently, a robotic jellyfish (Robojelly) was developed mimicking the morphology and propulsion mechanism of the *A. aurita* medusa species [9], [10]. The oscillating mode of propulsion utilized by jellyfish is not hydrodynamically efficient with a Froude efficiency ranging from 0.09–0.53 [11], [12], but the system as a whole is one of the most efficient. The cost of transport (COT) metric can be used to quantify the overall efficiency of a vehicle during transportation and is defined as:
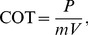
(1)where 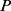 is the input power, 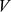 is the vehicle velocity and 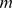 is the mass. The COT of jellyfish was found to be one of the lowest[13], [14]. The relatively low hydrodynamic efficiency is offset by the efficient metabolism and an elegant mechanical system. The design of a robotic jellyfish should attempt to incorporate all the relevant features that affect propulsion. Using Robojelly, controlled experiments can be conducted to understand the fundamental principles influencing the thrust production and overall energy efficiency. Physical parameters of the Robojelly can be varied systematically that would otherwise disrupt the physiological functions of the animal and therefore prevent any conclusive analysis. In this study, we provide the fundamental understanding of the section of the bell towards the tip that was found to play a significant role in the propulsive performance of the Robojelly [10]. This section is referred to as a flap or flexible margin. The effect of the flexible margin on the bell kinematics and starting vortex are the focus of this study.

## Methods and Materials

### 2.1 *Aurelia aurita* bell kinematics

**Figure 1:**
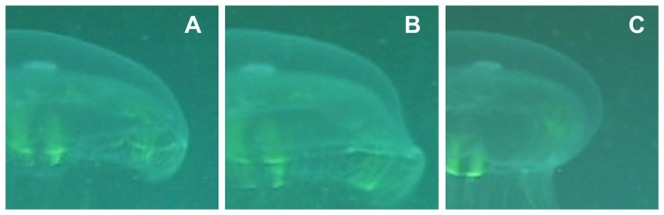
*Aurelia aurita* bell geometries. *Aurelia aurita* half bell profile in the (A) relaxed, (B) contracting and (C) contracted position. These images of *A. aurita* were taken during a flow characterization test using fluorescent dye. This test was conducted in ocean waters and the dye is seen as bright green in the images.

When designing the Robojelly, the morphology of an *A. aurita* measuring 6 cm in diameter was first replicated by digitizing the relaxed geometry of the animal. The bell kinematics was then analyzed in order to determine where to position the actuators. It was noticed that the bell margin did not follow the same deformation pattern as the rest of the bell. Figure 1 shows an *A. aurita* in the relaxed (a), contracting (b) and fully contracted states (c).

The exumbrella profile of the video in Fig. 1 was digitized over a full swimming cycle. The video was recorded at a frame rate of 29 fps and resolution of 720×480 pix. Every two to four images were processed during the swimming cycle with higher temporal resolution during contraction. Edge detection was done by importing the images in a computer aided design (CAD) software (Inventor, Autodesk) and manually selecting 10 to 15 points on each profile. It is critical to include margin points as accurately as possible in this process. A spline function was used to interpolate the points for a total of 401 points per profile. These pixel locations of the exumbrella were converted to polar coordinates and filtered using second order lowpass Butterworth filter. The points were then converted to Cartesian coordinates. The bell apex was selected as the middle point of the spline. The exumbrella points were then zeroed about the apex and normalized by a half exumbrella arclength in the fully relaxed position. Further information on the experimental procedure for tracking the deformation and image processing can be found in [9].

Properly defining the flap location is critical for analyzing its function. The flap can be defined as the region of the subumbrella that deforms passively during contraction. Chapman [14] demonstrated that subumbrellar muscles are located all the way to the bell margin which includes the flap region. It is unknown if the muscles in the flap region are actuated during contraction and if so, to what extent. Curvature is used to quantify the deformation along the arclength of the jellyfish bell. Curvature of the discrete exumbrella profiles were quantified using the definition of a circumcircle. A circumcircle is a circle passing through three vertices A, B and C of a triangle with sides of length a, b and c. The diameter (*d*) of a circumcircle is:
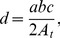
(2)where *A_t_* is the area of the triangle which can be found using Heron's formula: 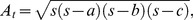
(3)where:
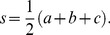
(4)


The curvature of the circumscribe circle is defined as:
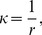
(5)where 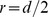 is the circumcircle radius. The curvature equation for a discrete curve in Cartesian coordinates becomes:
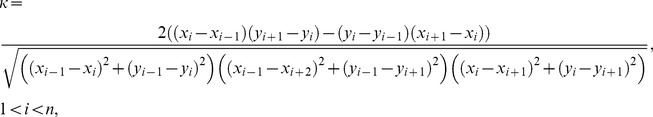
(6)where *i* is the point of an exumbrella profile and *n* is the total number of points for a given exumbrella profile.

### 2.2 BISMAC and Robojelly

**Figure 2:**
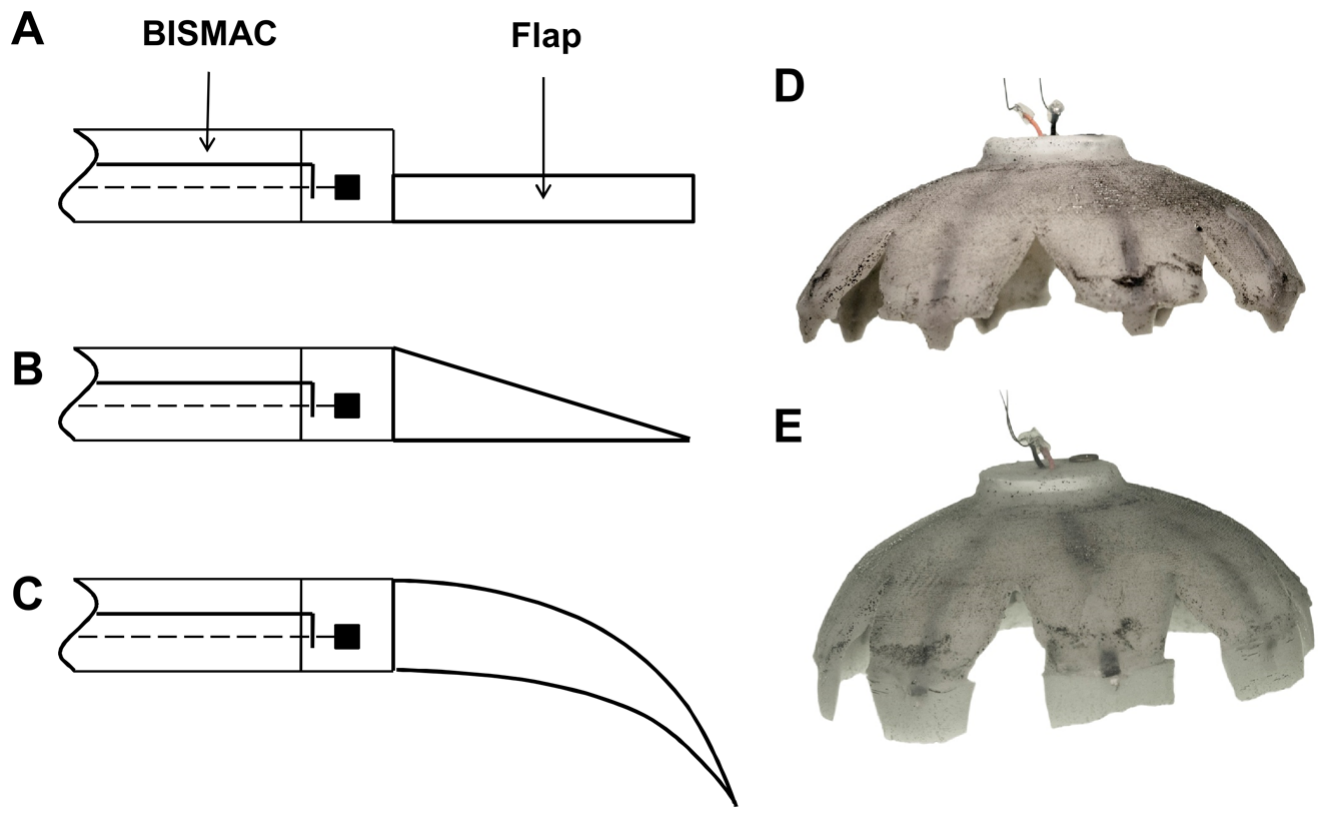
BISMAC actuators and Robojelly. Layout of BISMAC actuators with flaps having (A) rectangular, (B) tapered and (V) curved and tapered cross-section. Robojelly with (D) no flap (0% flap) and (E) Bio flap.

Before investigating the flap's role on hydrodynamics, it is essential to replicate similar kinematics as the *A. aurita*. Initial observations made from bell kinematics lead to the hypothesis that it can be recreated using a passive flexible margin since the flap seems to deform with flow resistance, see Fig. 1(b). This implies that no actuators would be required in the flap region. A bio-inspired shape memory alloy composite (BISMAC) actuator capable of providing high curvature change was utilized to test this hypothesis [9], [16]. The BISMAC actuator was optimized by varying the stiffness along its length to recreate a deformation profile similar to that of the *A. aurita*. The three flap designs used for testing the hypothesis are shown in Fig. 1. The BISMAC actuator dimensions were 0.6×1.3×17.5 cm and the flap was added at its tip. The first flap configuration tested had a rectangular cross-section of 0.3×1.3 cm, with length of 3 cm as shown in Fig. 2(a). The second flap configuration had a tapered cross-section and length of 3 cm as shown in Fig. 2(b). The tapering introduces a varying stiffness through the span of the flap. The third configuration had a taper and curvature which is more representative of the natural *A. aurita* flap and measured 4 cm in arclength, see Fig. 2(c). The BISMAC actuators with flap were tested in water by clamping at the base in a cantilever configuration. Reflective points distributed evenly along the side of the actuator and flap were tracked using image processing in Matlab. The different configurations of BISMAC actuators shown in Fig. 1 were all actuated under the same conditions.

When designing the Robojelly, the flap was left out of the active section that contains BISMAC actuators. The flap can be added to the vehicle separately allowing the analysis of different configurations. Figure 2(d) and (e) shows the Robojelly with and without flap. It should be noted that the Robojelly bell is segmented as opposed to uniform as is the case of *A. aurita*. The segmentation was made to alleviate bell folding. The flap added to Robojelly in Fig. 2(e) is called the Bio flap which is designed to replicate the same length, taper and curvature as the natural flap. The Robojelly and BISMAC flaps were made out of Ecoflex 0010 silicone from Smooth-On. This silicone has a density of 1040 kg m^−3^ and shear modulus of 1071.67 Pa. Further information on the silicone's mechanical properties can be found in [17] and a thorough description of the Robojelly design can be found in [10].

Both robot configurations were tested for their swimming performance. The vehicles were installed in a water tank with static water and were allowed to swim upwards. The robots were both actuated using a resistance feedback controller described in [10]. The controller sends a peak current of 1.5 A at the beginning of actuation and then maintains deformation with a current of 0.65 A. The vehicles were initially in a state of sinking to demonstrate that any thrust produced was from the propulsion mechanism and not the positive buoyancy. A thrust analysis described in [10] was used to quantify and compare the swimming performance of the Robojelly. Thrust was measured using position over time to quantify the vehicle momentum and buoyancy state. The hydrodynamic forces were approximated using a jetting model and empirical models were used for drag and added mass [11], [18].

### 2.3 TRDPIV

Flaps of constant cross-section with lengths of 33, 66, 100, 133, and 200% of the natural flap length were analyzed. The constant cross-section flap had a thickness of 0.25 cm. For the Robojelly with bell diameter of 16.4 cm, a 100% flap has the same proportional length as that of a 6 cm diameter *A. aurita* corresponding to 2.25 cm for the 16.4 cm bell diameter Robojelly. This was also compared to a “No flap” (0%) and “Bio flap” configuration which were tested in the swimming test. The bio flap has the same length as that of the 100% constant cross-section flap but the apex is tapered and curved. The Robojelly was first tested with the bio flap. Afterwards, the 200% flap with constant cross-section was tested on the same vehicle. The Robojelly was then tested for each flap length by cutting out sections until no flap was left.

**Figure 3:**
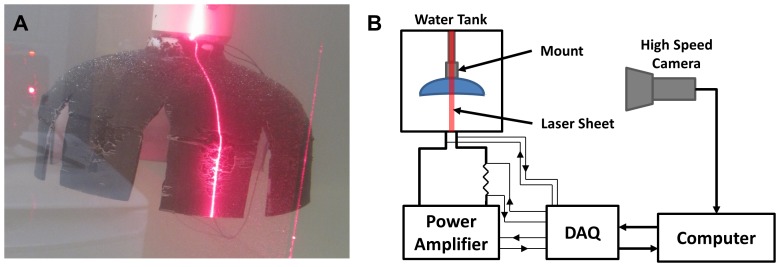
Experimental setup. (A) Robojelly with 200% flap setup on a mount in the water tank. The laser sheet is pointed at the bell. (B) Schematic of the TRDPIV test setup.

The Robojelly wake was analyzed using a time resolved digital particle image velocimetry (TRDPIV) system. The experimental setup consisted of a continuous laser sheet of 680 nm wavelength and 2W power (LaVision Inc.) aimed on the side of Robojelly as shown in Fig. 3. A high speed camera (Photron, Fastcam 1024 PCI) recording 1024×1024 pixel images at a rate of 400 FPS was set perpendicular to the laser sheet as shown in Fig. 3(b). The Robojelly was clamped at the apex inside a 122×46×51 cm water tank. Near neutrally buoyant glass particles with average diameter of 10 µm were used as flow tracers. The robot was actuated for 19 cycles before a full cycle was recorded for analysis. Preliminary actuation was necessary for the shape memory alloy (SMA) actuators to reach a thermal steady state. The thermal state of the SMA actuators affects the Robojelly's bell deformation [9], [10].

Cross correlation was performed on the high speed images to obtain the corresponding velocity fields (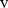). The data was processed using three passes of a Robust Phase Correlation (RPC) method [19]–[21] with image deformation [22] using window resolution of 32×32 vectors, window size of 64×64 vectors and a grid resolution of 4×4 pix. The mean error of this technique was estimated between 0.05–0.15 pix [19]. Proper Orthogonal Decomposition (POD) was used to post process the TRDPIV results to reduce the high frequency noise [23], [24]. The fundamental eigenmodes containing 90% of the total energy in the system were utilized in the analysis.

Vortex identification was done using a method described by Holden [25] which consists of two different identification methods and comparing the results of both. The first method is based on Sperner's lemma which is adapted for vortex identification in [26]. For this method, a first pass is done on the velocity field to label each velocity vectors in one of three equally spaced direction ranges. The second pass looks at the surrounding neighbors of every grid point and determines if all three direction labels are present. If so, the current point is identified as a vortex center. After identifying possible vortex locations with Sperner labeling, the 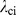 method was used to calculate the swirling strength of each possible vortex [27], [28]. The swirling strength is taken as the imaginary eigenvalue of 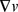. This is based on the fact that local streamlines can be represented as a function of real and complex eigenvalues. See [27] for more details and a visual representation of this method. The maximum swirling strength over the velocity fields was determined in a preliminary pass. Any potential vortex center having a swirling strength within 5% of this maximum was characterized as a vortex center. Erroneous vortex locations varying from the general trajectory of the starting vortex were manually removed.

### 2.4 Circulation

Once the vortex centers were identified, the vortex area *Α* was determined using the regions which fell within the 5% swirling strength threshold. Circulation of the starting vortex was computed using the line integral of the velocity field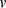, over the identified contour 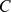
delimiting the vortex area:
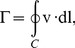
(7)where 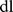 is the contour length vector. Vortex circulation and area were filtered using a second order Butterworth low pass filter. For scaling purposes, the bell margin was tracked as a function of time. Starting at rest, the margin position was detected manually using ImageJ. The distance traveled between each point was then calculated. The distances were smoothed using a second order Butterworth lowpass filter.

## Results and Discussion

### 3.1 Kinematics

#### 3.1.1 Aurelia aurita

**Figure 4:**
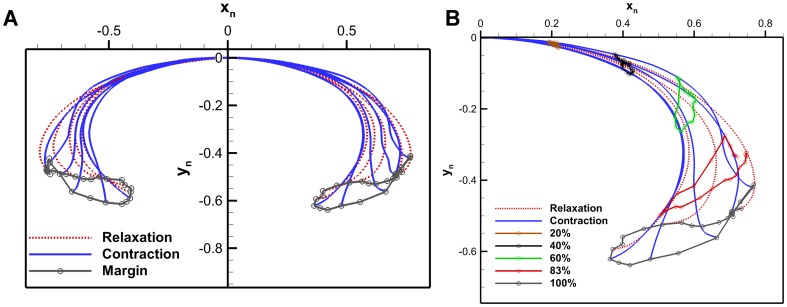
*Aurelia aurita* bell kinematics. (A) *Aurelia aurita* bell kinematics showing the exumbrella profiles over a full swimming cycle. The exumbrella profiles shown were selected arbitrarily for clarity. The margin trajectories are also shown for each side of the exumbrella profile. (B) Bell trajectories at selected points along the exumbrella arclength over a full swim cycle. The different percentages correspond to exumbrella arclengths from the apex to the margin. The 83% point corresponds to the flexion point and the 100% to the bell margin.

**Figure 5:**
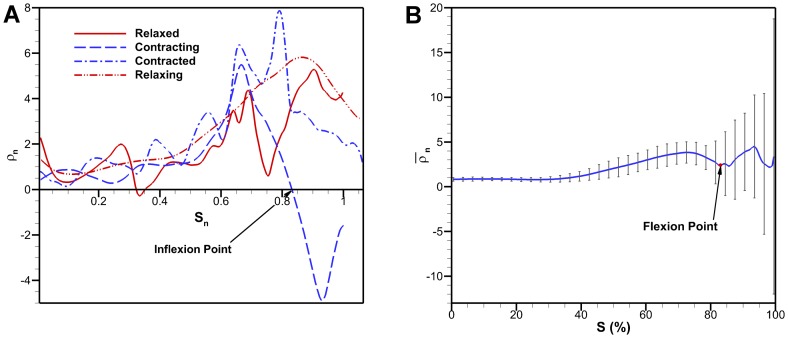
Exumbrella curvature. (A) Curvature as a function of exumbrella arclength from apex to margin for exumbrella profiles in different stages. The profiles shown cover the four main states of the bell kinematics: relaxed, contracting, contracted and relaxing. The curvature is normalized by exumbrella arclength in the relaxed state. (B) Average normalized curvature as a function of exumbrella arclength from apex to margin. The curvature is averaged over a full cycle which includes the left and right profiles of the *Aurelia aurita*. The curvature is normalized by exumbrella arclength in the relaxed state. The flexion point was found to be located at an arclength of 83% from the apex.

Digitized *A. aurita* profiles for a full cycle are shown in Fig. 4(a). The flap can be seen most distinct during the middle of the contraction. Curvature for selected profiles in the relaxed, contracted, relaxing and contracting configurations are shown in Fig. 5(a). The contracting profile shows a different curvature pattern than the others with a negative curvature towards the bell margin. The point at which curvature of the exumbrella profile goes from positive to negative is a possible lower bound of the flap. This location is called the inflexion point, defined as the location where a curve goes from positive curvature to negative curvature or vice-versa. Starting from the apex, the flap upper bound is simply the bell margin but several methods were considered to determine the location of the flap lower bound. The inflexion point location varies depending on the deformation magnitude. A stronger contraction will result in larger flap deformation and change the inflexion point location. Therefore, this must also be considered when determining the flap lower bound location. From this point onward, the lower bound location of the flap will be referred to as the flexion point.

The flexion point can be determined by finding the point associated with the minimum curvature or largest negative curvature over a full cycle. The largest negative curvature occurs during contraction. However, this method does not take into account the variation in curvature over time. The average flexion point location during strong contraction can be determined. A strong contraction can be defined as the region where the largest minimum curvature falls within a specified threshold. This method leads to a somewhat arbitrary selection of profiles determined by the threshold values. The chosen method calculates the time average curvature for individual arclength locations and the associated standard deviation during an entire swimming cycle. In addition, the curvature for both the left and right side of the exumbrella profiles were averaged. The resulting average curvature as a function of exumbrella arclength is shown in Fig. 5(b). The flap location was selected as the location where the average curvature reaches a local minimum with value of 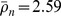 and where the standard deviation starts to diverge with value of 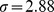. This corresponds to a flexion point location of 83% making the flap 17% of the exumbrella profile. If the transition location between the active and passive bell section was known, this could be a more direct method of determining the flexion point location but as previously mentioned, that location is unknown for the *A. aurita*.

The bell margin follows looping trajectory with outer path during contraction and inner path during relaxation as shown in Fig. 4. The flexion point follows an inner path during contraction and outer path during relaxation, as shown in Fig. 4(b). This is also true from the apex until approximately 90% of exumbrella arclength where the contraction and relaxation paths become overlaid through most of the swim cycle. During relaxation the flap undergoes a “roll out” motion as it abducts which reduces the bell area normal to the bell motion.

Looping in the bell margin trajectory is of significant importance because the range of motion, velocity and acceleration are highest in the flap region. This can be visualized with a half cross-section of the bell rotating about a pivot point located at the apex. The moment arm amplifies the magnitude of the hydrodynamic forces in that region. The outer path during contraction is favorable to produce thrust. The length of the moment arm during the outer path will affect circulation of the starting vortex as shown in Section 3.4. The inner trajectory during relaxation is favorable to reduce drag on the vehicle and resistance to the passive relaxation.

The flap has previously been modeled as a body passively pivoting at the bell periphery [29], [30]. Wilson and Eldredge [30] have modeled a two-dimensional solid body structure which loosely resembles a jellyfish. The body consisted of solid elliptical bodies connected through hinges. They explored the effects of actively and passively controlling the different bodies with prescribed or passive motion at the hinges. They found that passively controlling the marginal bodies increased performance. McHenry and Jed [29] modeled the *A. aurita* swimming mechanism as jetting and paddling. Thrust calculation with the jetting model based on [11] overestimated the measured *A. aurita* thrust for larger rowers. The paddle model approximated the flap as a flat plate oriented perpendicular to the flow and calculated thrust produced by the flap only. Thrust produced by paddling significantly underestimated the measured speed by an order of magnitude. Though the jetting model gave a better representation of the *A. aurita* hydrodynamics, it does not take into account the physics from the starting and stopping vortices observed around the flap and there interaction with each other [2], [6], [31].

#### 3.1.2 BISMAC

**Figure 6:**
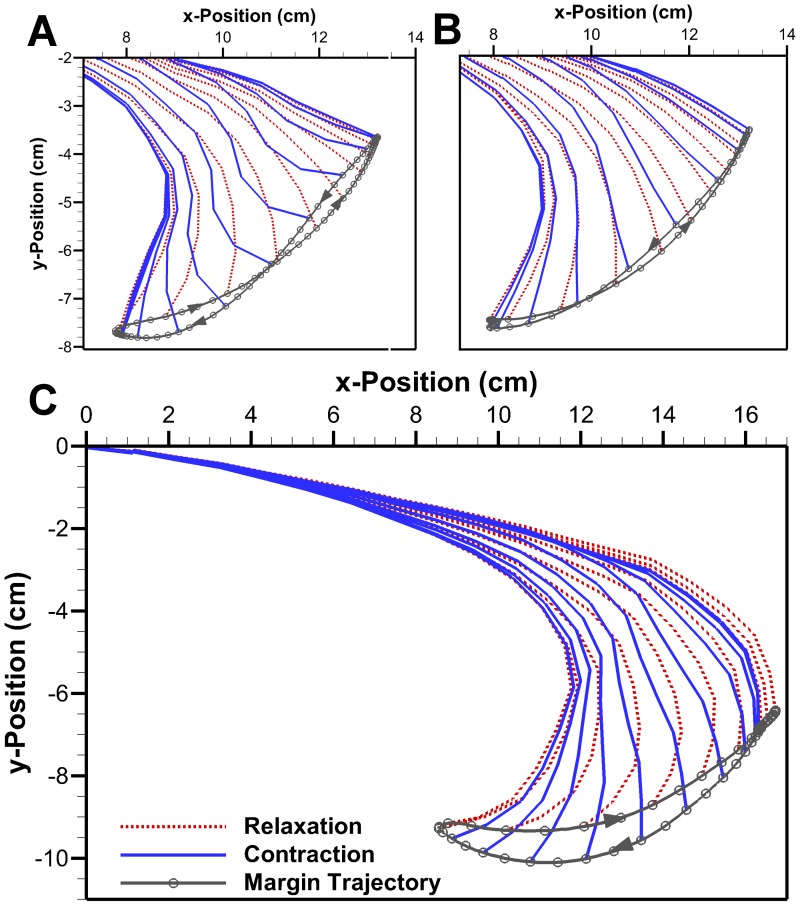
BISMAC kinematics. BISMAC actuator profile deformation with (A) constant, (B) tapered and (C) curved and tapered cross-section. Margin trajectories are shown with arrowheads indicating time progression. Profiles were down sampled in this figure for clarity.

The BISMAC actuator profiles and margin trajectories over a full cycle are plotted in Fig. 6 for the different flap configurations shown in Fig. 2(a). The constant cross-section flap which was the least stiff configuration had a margin trajectory resembling the number “8”. The beginning of the contraction starts with an inner path and transitions about half way into an outer path. The same is true for the relaxation phase. The constant cross-section flap does show a negative curvature during contraction as seen with the *A. aurita*. The deformation profile of the tapered flap in Fig. 6(b), does not vary much during contraction and relaxation. The increased flap stiffness prevented higher curvature changes during relaxation. Adding a curved profile caused the flap to already have a curvature during relaxation. Contraction of the curved and tapered flap shows an extension of the flap due to water resistance in Fig. 3(c). The margin trajectory shows an outer path during contraction and an inner path during relaxation as achieved by the *A. aurita*.

These results show that it is indeed possible to replicate the important characteristics of the *A. aurita* flap kinematics using a passive flap. In terms of bio-inspired vehicle design, limiting the number of actuators reduces the complexity of the design dramatically and ultimately can lead to more efficient vehicles. Judging from the BISMAC kinematic results, it can be hypothesized that the flap muscles of the *A. aurita* do not actuate much, if at all, during straight swimming. This is based on the fact that similar kinematics are recreated using a passive flap. The Robojelly design assumes that the flexion point is where the transition from active to passive occurs and this greatly simplifies the architecture. The role of circular muscles in the flap could be justified during a turning maneuver where the bell needs to achieve an asymmetric configuration and requires certain parts of the flap to stay stiff.

#### 3.1.3 Robojelly

**Figure 7:**
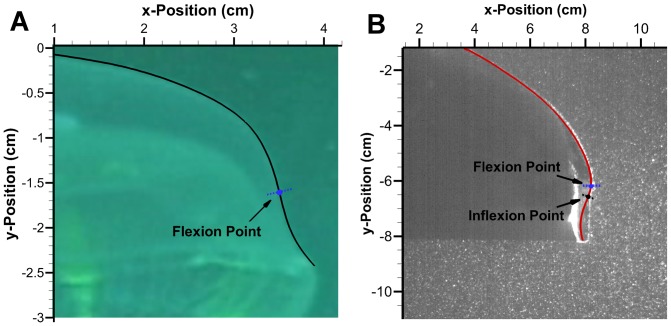
Flap location. Superimposed exumbrella profile with flexion point location during contracting for the (A) *Aurelia aurita* and (B) Robojelly. The Robojelly inflexion point in (B) is for the single contraction profile shown.

**Figure 8:**
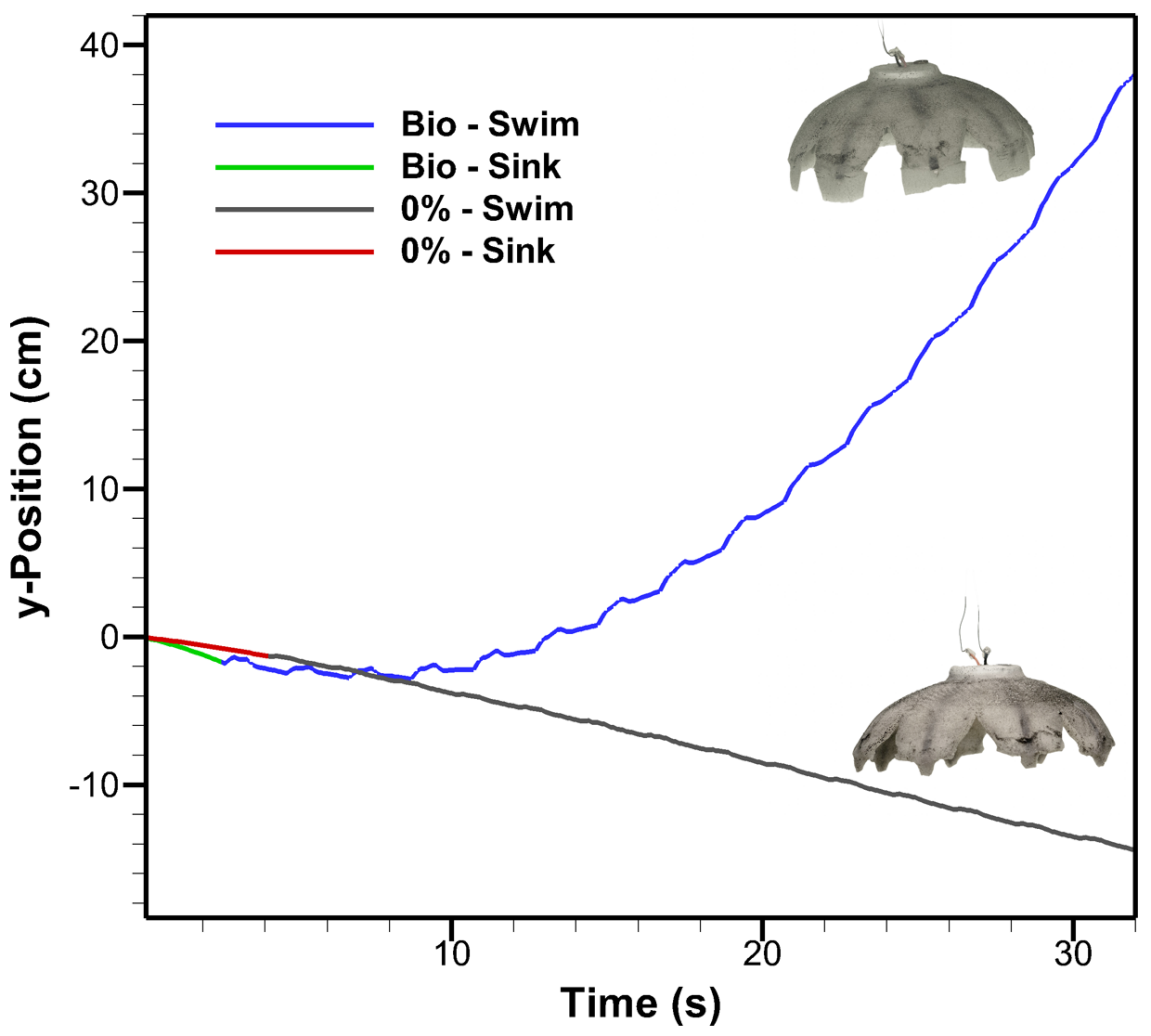
Robojelly swimming performance. y-Position as a function of time for Robojelly with and without the bio flap during vertical swimming. The initial sinking state of the robot is shown along with the swimming state. The robot was not actuated during the initial sinking state.

The bio flap on the Robojelly matches the 17% exumbrella arclength found in the *A. aurita*. A visual inspection of the exumbrella profiles during contraction Fig. 7(a) and 7(b), shows discrepancy between the *A. aurita* and Robojelly. This is mainly due to an increase in local stiffness due to the manufacturing constraints at the actuator tip. The actuator tip has a rigid attachment for the SMAs as seen in Fig. 2(a), which effectively decreases the flap flexibility in that region. As a result, the inflexion point occurs farther along the exumbrella arclength for a given contraction profile. Despite the differences in flap kinematics, a significant improvement in swimming performance was observed with the addition of a flap as shown in Fig. 8. A thrust increase of 1340% was calculated in [10] from the 0% flap configuration to the bio flap configuration.

#### 3.1.4 Robojelly kinematics

**Figure 9:**
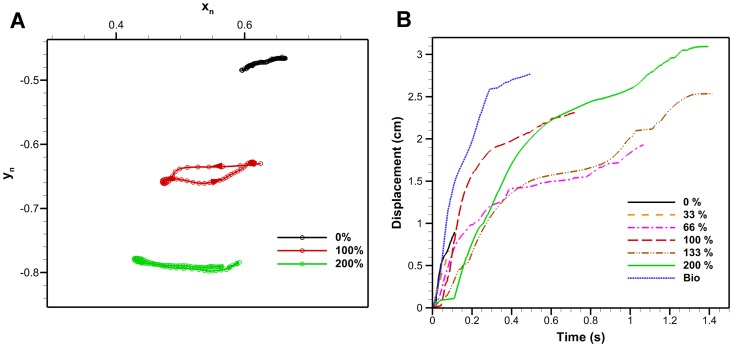
Robojelly kinematics. (A) Robojelly margin trajectories for different flap configurations. The trajectories are for a Robojelly normalized at its apex (position (0, 0)). Arrows indicate margin direction. (B) Bell margin displacement as a funciton of time during actuation for the different flap lengths. Displacments are shown from the beginning of actuation till the end of motion in the negative x-direction.

Figures 4 and 9(a) show that the Robojelly underperformed the *A. aurita*'s bell displacement. For the 100% constant cross-section flap, displacement in the x-direction is 60% less than that for the *A. aurita* while displacement in the y-direction is 85% less. In addition to total displacement differences, the trajectories also differ. The 100% case has a larger distance between its contraction and relaxation path but has an outer path during contraction as opposed to outer path with the *A. aurita*, see Fig. 9(a). The 0% and 200% flaps have near constant paths during contraction and relaxation.

Flap displacement is calculated as the arclength traveled by the flap tip and is shown as a function of time for all flap configurations in Fig. 9(b). The longer peak time with increasing flap length can be attributed to the the flap structural dynamics and SMA performance. The longer the flap, the more bending it undergoes. As a result, a larger lag occurs at the margin relative to the actuator tip (flexion point).

Figure 9(b) shows that the bio flap and 100% flap were actuated at different rates and covered different distances. The actuation difference between these two flap configurations is mainly related to actuator performance. SMA actuation performance can degrade because of overheating, shakedown, stretching and slipping at the connections. The bio flap was tested first which means the actuators were in their best condition. The constant cross-section flap at 200% was then tested followed by the 133% and so on until the 0% flap configuration. Additionally, the added mass from the larger flaps prevented a fully relaxed position causing the actuation cycle to be smaller.

### 3.2 Starting Vortex Circulation

**Figure 10:**
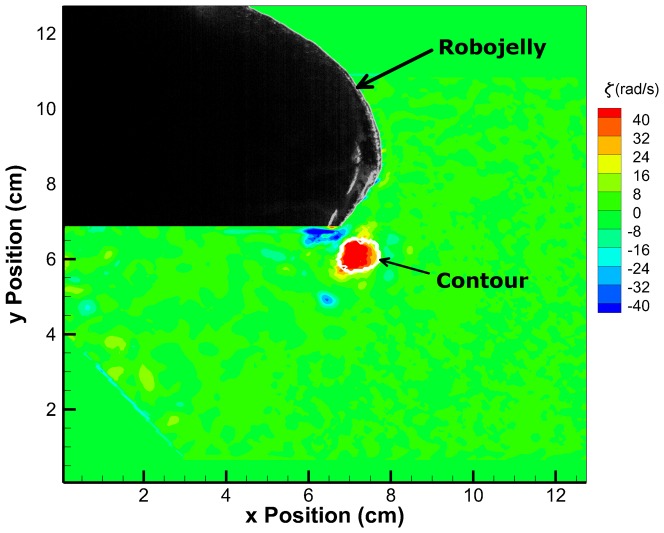
Vorticity field. Vorticity field of the Robojelly with bio flap at 0.2

**Figure 11:**
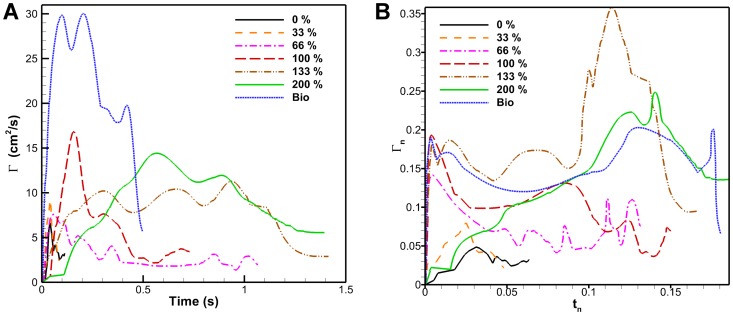
Dimensional and non-dimensional circulation. (A) Robojelly circulation as a function of time for different flap configurations. (B) Non-dimensional circulation as a function of time scaled by orifice diameter.

An example vorticity field created by the Robojelly with bio flap is shown in Fig. 10. Vorticity was calculated using:
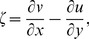
(8)where 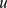 and 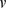 are the 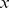 and 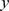 components of the velocity field respectively. This figure also shows the starting vortex and contour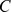 used for calculating circulation. Dimensional circulation as a function of time is shown in Fig. 11(a) for different flap configurations. The constant cross-section flaps have a general increase in peak vortex strength as a function of flap length. The time it takes for the vortex to reach peak circulation also increased with flap length. The bio flap had a much higher circulation then the other flaps though it was of the same length as the 100% flap. This is partly due to the deterioration of the actuator performance as stated in the previous section. To determine the underlying principles of flap length, varying parameters can be accounted for by making circulation and time non-dimensional. The Robojelly flap was scaled using two different methods.

The first method approximates the Robojelly as an axi-symmetric body which ejects water from a varying orifice. This is similar to work on varying orifice pistons where circulation and time are scaled as a function of piston velocity and the orifice diameter is scaled as a function of time [32], [33]. Robojelly has a varying diameter but not a moving piston. Water displacement on the Robojelly is due to actuation of the bell or walls which contract or reduce the diameter of the Robojelly. The non-dimensional equations in [32] can be modified to take into account water displacement due to wall deformation:
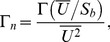
(9)

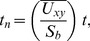
(10)where 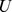 is the magnitude of the flap tip velocity 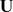 and 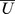 is the time average velocity:
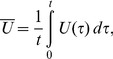
(11)where 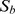 is the arclength of the exumbrella from the bell apex to the flap tip. The resulting non-dimensional circulation is shown in Fig. 11(b). The results show a wide variability of the circulation peaks and no apparent scaling.

**Figure 12:**
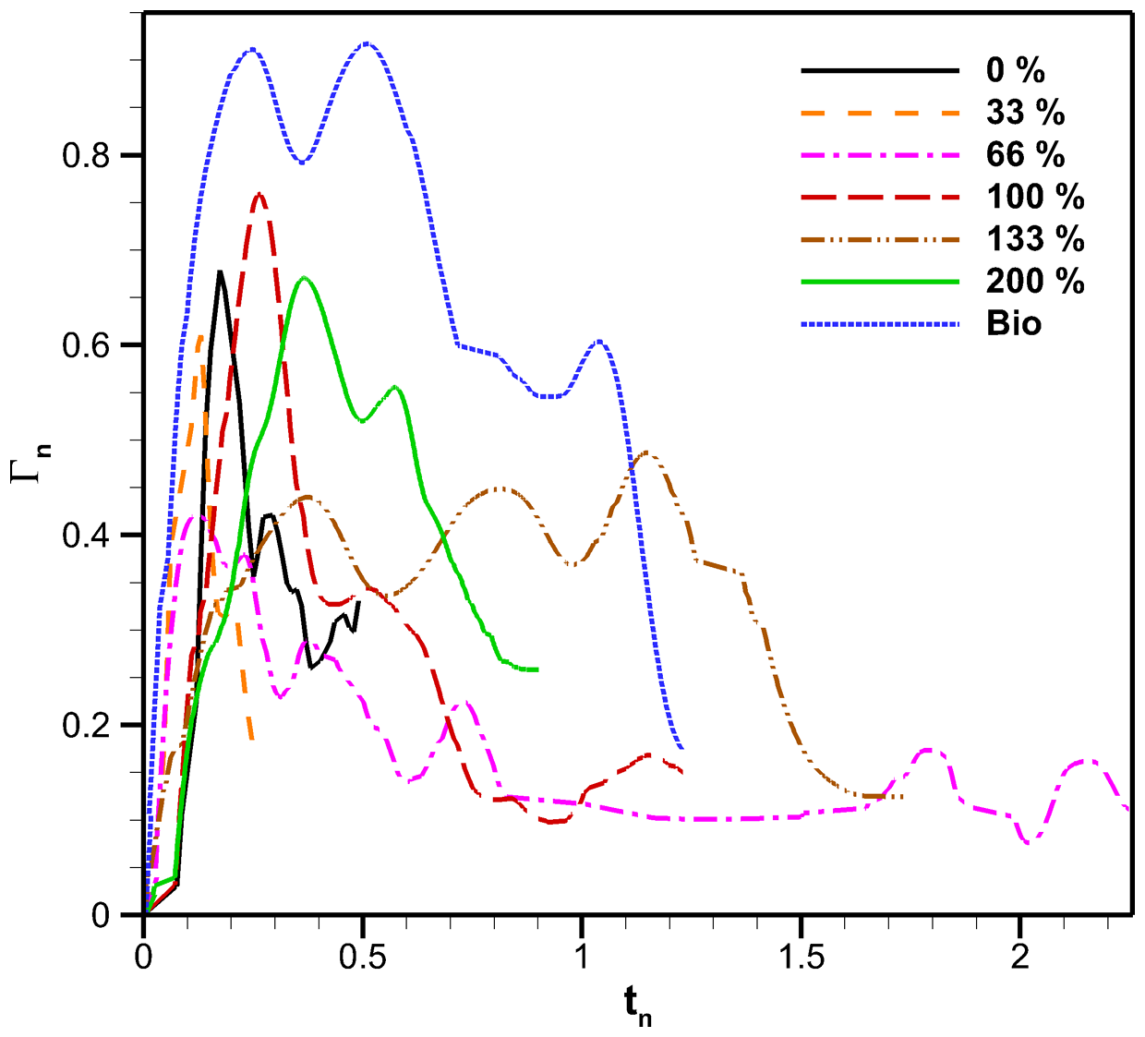
Non-dimensional Robojelly circulation using arclength. Non-dimensional circulation scaled by bell arclength form apex to bell margin, as a function of non-dimensional time for different flap configurations.

The second method for scaling circulation analyzes the Robojelly as a set of pitching panels distributed circularly about the bell apex. The hinge location is approximated by the point where the highest rate of deformation as a function of arclength occurs. The hinge location was found to be at 1.5 cm exumbrella arclength above the flexion point. The length 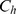 is defined as the arclength between the hinge location and the flap margin. Buchholz and Smits [34] have demonstrated the importance of panel aspect ratio (AR) which is the span over chord of the panel, and the ratio A/S which is the panel maximum amplitude over span. Green and Smits [35] have found that the coefficient of pressure 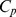 of a pitching panel was best scaled by:
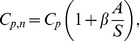
(12)where 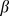 is an arbitrary constant. It was found that a value of 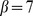 best scales the coefficient of pressure and coefficient of thrust. Buchholz et al. [36] expanded this method to the scaling of circulation for pitching panels: 
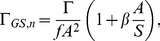
(13)where 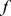 is the frequency of actuation. This scaling method therefore takes into account the geometry and kinematics of the pitching panel. This method can be adapted to the Robojelly flap as follows:
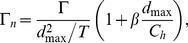
(14)

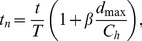
(15)where 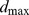 is the maximum distance traveled by the flap margin during contraction before it starts moving backwards, and 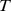 is the time taken from the beginning of actuation to reach 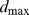. It should be noted that the variable 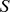
in eqs. 12 and 13 represent the span of a pitching panel which is perpendicular to the primary flow. In comparison, the variable 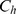 in eqs. 14 and 15 represent the chord of the flap which is parallel to the primary flow. The resulting non-dimensional circulation as a function of non-dimensional time for the different flap configurations are plotted in Fig. 12.

The chord dimension best scales circulation here as opposed to the span dimension found for pitching panels [35], [36]. This is likely due to the circumferential distribution of flaps that act as pitching panels in close proximity, thereby reducing the influence of spanwise vortices. The starting vortex formed by the Robojelly flap is a tip vortex at the bell margin due to pitching motion. In comparison, the natural jellyfish creates an excess pressure in its subumbrella that causes flow over the bell margin and forms a starting vortex.

**Figure 13:**
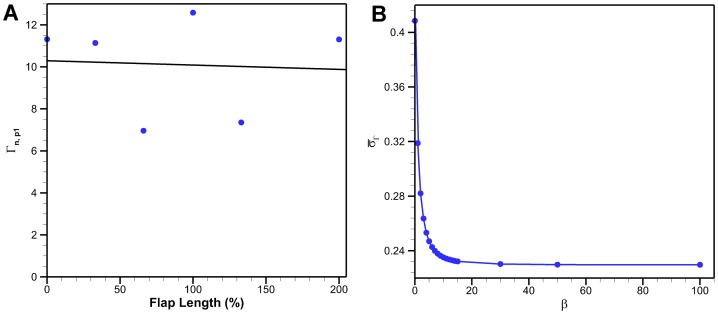
Non-dimensional circulation results. (A) Non-dimensional circulation of the first peak as a function of flap length for β = 15. This does not include the bio flap. The normalize standard deviation is 0.23. (B) Normalized standard deviation of first peak circulation as a function of β.

The first peak of non-dimensional circulation as a function of flap length is shown in Fig. 13(a). The bio flap is excluded from these results since it has a different geometry. The standard deviation of the first peaks is normalized by the mean non-dimensional first peak circulation:
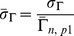
(16)


Figure 13(b) shows that 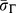 converges to 0.23 as 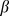 increases. Buchholz et al. [36] found that the rigid pitching panels had a normalized standard deviation of 0.15 and 0.04 for 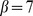 and 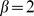 respectively. The higher standard deviation of flaps compared with the rigid pitching panels is likely related to the added flexibility. Flexibility causes the flap base and tip to move at a different phase. It also allows the flap to assume different curvature profiles throughout actuation. The longer the flap, the larger the geometric change during an actuation cycle.

The non-dimensional circulation results of Fig. 12(a) show that the bio flap follows a different scaling trend then the constant cross-section flaps. The bio flap is curved and tapered which creates a different deformation profile upon actuation. This leads to the conclusion that initial flap geometry which affects stiffness and therefore its transient shape also has an effect on vortex circulation. A better understanding of the effects of curvature and varying curvature as a function of time is necessary to scale circulation with a smaller standard deviation. In addition, an understanding of flap compliance for a given stiffness and geometry is necessary to know its instantaneous geometry. The 133% flap exhibits a significantly different pattern than the other constant cross-section flaps. This could be related to three dimensional instabilities or not properly resolving the full dynamics of the flow which would in turn have compromised the vortex identification process and would result in lower circulation amplitude.

The contribution of each term in Eq. 14 was analyzed by finding 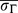 for both terms individually. When only considering the first term, which means neglecting the flap length, the normalized standard deviation was 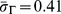. The second term of Eq. 14 which includes flap length and kinematics, without the arbitrary constant 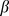 resulted in 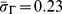. The dominance of the second term justifies neglecting the first term and Eqs. 14 and 15 become:
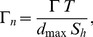
(17)

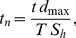
(18)


The comparison between the scaling results of the varying orifice model and pitching panel model show that the Robojelly flow is better described using the pitching panel model. The varying orifice model takes into account the varying orifice diameter and the water velocity at the outlet. This is a form of the jetting model often used to represent the propulsion mechanism of jellyfish [11]. The jetting model quantifies thrust production of a jellyfish by approximating its wake as a slug of water ejected during contraction. It does not take into account the time dependent geometry variation of the body and its effect on the water structure being ejected. This method is ideal for a rigid cylinder with piston apparatus and gives a good approximation for jetting jellyfish that have a prolate geometry and undergo small orifice deformation during actuation. However, rowing jellyfish are more oblate and undergo large orifice diameter changes during contraction and therefore significant changes in their overall geometry. The pitching panel method better accounts for the geometry of the body by approximating the bell as a panel rotating about a hinge. The starting and stopping vortex interaction of a rowing jellyfish is critical to its propulsion [2], [12]. The interaction between both vortices is dictated by their location and strength which in turn depend on the geometry and kinematics of the bell. Thus this work suggests that a pitching panel model would provide better insights on the vortex formation, interaction and therefore thrust production of rowing jellyfish.

The relaxation phase is not taken into account by the jetting model. Dabiri et al. [37] added to the jetting model by including the force created during relaxation based on the strength of the stopping vortex. However, the vortex circulation and area are roughly approximated based on the slug model [38] and oral cavity respectively which were calculated as a function of margin diameter. Though performance was not directly compared with an animal, the model gave a good relationship with expected medusan morphology. Gemmell et al. [14] found that thrust was also produced during relaxation which shows the importance of accounting for the relaxation phase of rowing jellyfish. The pitching panel approach better lends itself for modeling the relaxation phase since the vortex formation can be calculated based on the geometry and kinematics of the panel and the surrounding flow properties. Panels with geometries varying as a function of span and time will need to be used for modeling the propulsion of rowing jellyfish. This would take into account the effects of a flap as part of a single panel geometry as opposed to a separate structure. Finally, the panel model will need to be expanded to an axisymmetric geometry for uniform bells. Though some jellyfish such as the *Cyanea capillata* are naturally segmented, most jellyfish have a uniform bell [39], [40].

## Conclusions

The flap kinematics of the *A. aurita* revealed some important parameters. Curvature changes from positive to negative and the margin trajectory follows an outer path during contraction and inner path during relaxation. This can be achieved with a flexible passive structure and alleviate the need for a separate set of actuators. Flap kinematics can be modified by the geometry, material composition, actuation speed and acceleration. The fact that the flap can be recreated artificially by a passive structure raises the following question: “Do *Aurelia aurita* actuate their flap during swimming?” It is known that muscles are located inside the flap but the extent at which they actuate during bell contraction is unknown [15]. Further research in the animal's histology will be required to answer this question. This would also help determine if the flexion point correlates to the transition between a passive and active section. An important aspect to consider for replicating flap kinematics of the *A. aurita* is axi-symmetry. The axi-symmetry of the natural flap causes additional structural integrity which will affect the kinematics. Also, the flow escaping around the BISMACs and Robojelly bell segments is trapped in the case of the *A. aurita* and will cause additional resistance and therefore additional flap deformation.

Uncertainty calculation for data obtained using PIV is very intricate and depends on factors such as user inputs, flow characteristics and experimental setup. A detailed uncertainty analysis is beyond the scope of this paper and does not affect the conclusions drawn from the results. Future work will address this limitation and will utilize recent advancement in tools for assessing uncertainty of PIV measurements [41], [42].

Future work on modeling of rowing jellyfish should account for the flap and the rest of the bell. It should consider both jetting and vortex formation. Also, the role of a flap in positioning stopping and starting vortices should be addressed. The interaction between stopping and starting vortices is known to play a crucial role in jellyfish propulsion [2], [12]. Since flaps create a significant bell geometry change, it will affect positioning of the starting and stopping vortices and therefore affect the overall performance. Two methods were used to scale the Robojelly circulation. A pitching panel representation of the segmented bell gave a better scaling of circulation over the jetting with varying orifice representation. This postulates that it is beneficial to use a pitching panel theory to better understand the fundamental propulsion mechanisms of rowing jellyfish. In order to confirm this hypothesis, the pitching panel theory will need to be applied to an axisymmetric bell and compared to the jetting model. In this study, the flaps were approximated as rigid panels for scaling. The effect of flexibility on pitching panels is still poorly understood and will require further research.
